# Economic value of finotonlimab plus bevacizumab versus sorafenib for first-line treatment of unresectable hepatocellular carcinoma in China and the United States

**DOI:** 10.3389/fpubh.2026.1781790

**Published:** 2026-07-06

**Authors:** Yulong He, Qinling Jiang, Xia Pan, Lin Deng, Xinrong Hu, Zhijian Yang, Wenwang Lang

**Affiliations:** 1 Department of Oncology, Nanxishan Hospital of Guangxi Zhuang Autonomous Region, Guilin, China; 2 Department of Hepatobiliary Surgery, Nanxishan Hospital of Guangxi Zhuang Autonomous Region, Guilin, China; 3 Department of Pharmacy, Nanxishan Hospital of Guangxi Zhuang Autonomous Region, Guilin, China

**Keywords:** bevacizumab, cost-effectiveness, finotonlimab, hepatocellular carcinoma, Markov model, sorafenib

## Abstract

**Background:**

Finotonlimab combined with bevacizumab demonstrated superior progression-free survival and overall survival compared with sorafenib in patients with unresectable hepatocellular carcinoma. However, its economic value in different healthcare systems has not been fully characterized. This study evaluated the cost-effectiveness of finotonlimab plus bevacizumab from the perspectives of U.S. payers and the Chinese healthcare system. A Markov state-transition model was constructed using clinical efficacy data from a phase 3 randomized controlled trial.

**Methods:**

Health state utilities were obtained directly from trial data, and cost inputs were derived from published sources and country-specific charge databases. The outcomes included quality-adjusted life years (QALYs), incremental cost-effectiveness ratios (ICERs), incremental net health benefits (INHBs), and incremental net monetary benefits (INMBs). Scenario analyses, deterministic and probabilistic sensitivity analyses, and price simulations were performed to assess the robustness and cost-effectiveness across predefined willingness-to-pay (WTP) thresholds.

**Results:**

In the base-case analysis, the ICER of finotonlimab plus bevacizumab compared with sorafenib was $27,505.33 per QALY in China and $138,055.71 per QALY in the U.S., both below the corresponding WTP thresholds of $40,354.27 and $150,000 per QALY, respectively. The associated INHBs were 0.26 QALYs in China and 0.07 QALYs in the U.S., with INMBs of $10,523.82 and $10,684.73, respectively. Probabilistic sensitivity analysis indicated that combination therapy was cost-effective in 91.43% of simulations in China and 58.33% in the United States. Price simulation analyses suggested that finotonlimab would remain cost-effective in the U.S. setting when priced below $9,157.84 per 200 mg.

**Conclusion:**

From both Chinese and U.S. payer perspectives, finotonlimab plus bevacizumab represents a cost-effective first-line treatment strategy for unresectable hepatocellular carcinoma. These findings offer quantitative support for pricing and reimbursement decisions related to this emerging immunotherapy combination targeted therapy.

## Introduction

Liver cancer remains a major contributor to the global cancer burden, ranking among the most frequently diagnosed malignancies and leading cause of cancer-related mortality worldwide. In 2022, an estimated 865,000 new cases were diagnosed globally, with approximately 757,948 deaths attributed to liver cancer (1). Hepatocellular carcinoma (HCC) is the predominant histological subtype, accounting for approximately 75–85% of primary liver cancers (2, 3). The disease burden is unevenly distributed, with China alone accounting for 42.4% of the global liver cancer cases. In parallel, the U.S. has experienced a sustained increase in both HCC incidence and mortality in recent decades, with the age-standardized incidence rate rising by 2.8% between 2017 and 2021 (4). In parallel, the U.S. has experienced a sustained increase in both HCC incidence and mortality in recent decades, with the age-standardized incidence rate rising by 2.8% between 2017 and 2021 (2, 5).

Owing to its insidious onset, HCC is frequently diagnosed at intermediate or advanced stages, with more than 70% of patients presenting beyond the window for curative treatment (2). Advances in systemic therapy, particularly the development of targeted agents and immune checkpoint inhibitors (ICIs), have reshaped the therapeutic landscape for advanced diseases. The introduction of sorafenib revolutionized the treatment paradigm for advanced HCC, transitioning the disease from untreatable to manageable, significantly prolonging survival and improving quality of life, ushering in the era of precision treatment for liver cancer and providing important benchmarks for subsequent novel drug development (6). While early studies demonstrated that single-agent ICIs produced higher durable response rates than sorafenib, these agents did not confer a statistically significant overall survival (OS) benefit (7). This limitation prompted the exploration of combination strategies, including ICIs combined with bevacizumab, ICIs paired with tyrosine kinase inhibitors (TKIs), and dual immune checkpoint blockade. As a result, multiple regimens incorporating programmed cell death protein 1 (PD-1) inhibitors, such as atezolizumab, sintilimab, camrelizumab, toripalimab, and penpulimab (8–13), in combination with anti–vascular endothelial growth factor (VEGF) therapy or TKIs have emerged as the leading treatment options. Although regulatory approval remains pending for several of these combinations, contemporary clinical guidelines increasingly recommend ICI-based combination regimens as the first-line therapy for advanced HCC.

Finotonlimab (SCT-I10A) is a humanized immunoglobulin G monoclonal antibody directed against the PD-1 pathway (14). Preclinical investigations and early clinical studies have demonstrated its antitumor activity and favorable safety profile for multiple malignancies (15, 16). More recently, evidence from a multicenter, open-label, randomized controlled trial (RCT) indicated that finotonlimab in combination with bevacizumab significantly outperformed sorafenib as a first-line treatment for HCC, yielding clinically meaningful improvements in both progression-free survival (PFS) and OS (17). Importantly, the safety profile of this dual-agent regimen was consistent with expectations for comparable therapies, with no unexpected or unmanageable adverse events reported.

In China, domestically developed PD-1 monoclonal antibodies generally have lower acquisition costs than all PD-L1 inhibitors and imported ICIs. The introduction of finotonlimab is expected to intensify market competition, potentially reducing treatment expenditure and improving patient access to effective therapies. Finotonlimab has received regulatory approval from the National Medical Products Administration (NMPA) of China and is anticipated to enter international markets. However, the absence of established pricing information in the U.S. introduces uncertainty into economic evaluations. Against this background, the present study using Markov model aimed to evaluate the cost-effectiveness of finotonlimab plus bevacizumab compared with sorafenib as first-line therapy for advanced HCC from both the U.S. payer and Chinese healthcare system perspectives, with the objective of informing future pricing and reimbursement decisions.

Markov model is widely used in tumor pharmacoeconomic analyses (18). A key challenge in Markov model construction is the calculation of transition probabilities, whose parameters are typically not directly obtainable and require calculation and adjustment based on clinical literature or epidemiological data. Obtaining time-varying transition probability parameters (dynamic models) through survival analysis is a feasible solution. Although applying survival analysis to the calculation of Markov model parameters for pharmacoeconomic evaluation has limitations, it remains one of the effective and feasible methods for addressing time-dependent transition probabilities in dynamic Markov models, particularly in oncology pharmacoeconomics.

## Materials and methods

### Patient enrollment and treatment strategies

This economic evaluation was conducted in accordance with the Consolidated Health Economic Evaluation Reporting Standards (CHEERS) guidelines (19). The modeled population reflected patients enrolled in the phase 3 RCT (17), including adults aged 29–79 years with histologically confirmed HCC that was locally advanced, unresectable, or metastatic.

Patients received either finotonlimab (200 mg intravenously on day 1) in combination with bevacizumab (15 mg/kg intravenously on day 1) or sorafenib (400 mg orally, twice daily). All the treatments were administered in 21-day cycles and continued until radiographic disease progression, unacceptable toxicity, or voluntary discontinuation. Post-progression treatment strategies followed recommendations from the National Comprehensive Cancer Network (NCCN) (20) and the Chinese Society of Clinical Oncology (CSCO) (21) guidelines for HCC, consistent with the treatment pathways applied in a phase 3 trial (17). In the finotonlimab plus bevacizumab arm, subsequent therapy consisted of lenvatinib in both China and the U.S. or best supportive care (BSC). In the sorafenib arm, the patients received sintilimab plus bevacizumab in China, atezolizumab plus bevacizumab in the U.S., or BSC.

Treatment-related adverse events were incorporated into the model, with a focus on grade 3 or higher events occurring in >3% of patients. These events included proteinuria, thrombocytopenia, leukopenia, hypertension, hypokalemia, and palmar–plantar erythrodysesthesia syndrome (Tables 1, 2).

**Table 1:** Key clinical input data.

Parameters	Baseline value	Range		Distribution	Reference
		Minimum	Maximum		
Survival model for OS					
Finotonlimab plus Bevacizumab	Mu = 2.782 Sigma = 1.273 *Q* = −0.798			Gengamma	(17)
Sorafenib	meanlog = 2.621 sdlog = 1.125			Log-Normal	(17)
Survival model for PFS	Survival model for PFS				
Finotonlimab plus Bevacizumab	Mu = 1.059 Sigma = 0.638 *Q* = −2.578			Gengamma	(17)
Sorafenib	Mu = 0.915 Sigma = 0.493 *Q* = −1.823			Gengamma	(17)
Drug cost, $/per cycle					
Cost of Finotonlimab	1,370.46	1,096.37	1,644.55	Gamma	Local charge, (30)
Cost of Bevacizumab	702.08	561.66	842.50	Gamma	Local charge, (30)
Cost of Sorafenib	268.92	215.14	322.70	Gamma	Local charge, (30)
Cost of Lenvatinib	155.10	124.08	186.12	Gamma	Local charge, (30)
Cost of Sintilimab	303.3	242.64	363.96	Gamma	Local charge, (30)
Cost of Atezolizumab	4,605.64	3,684.51	5,526.77	Gamma	Local charge, (30)
Cost of Regorafenib	49.71	39.77	59.65	Gamma	Local charge, (30)
Cost of Tislelizumab	352.03	281.62	422.44	Gamma	Local charge, (30)
Cost of the laboratory test	57.83	46.26	69.40	Gamma	(31)
Enhanced CT	91.14	72.91	109.37	Gamma	Local charge, (32)
Cost of end-of-life	1,839.00	1,471.20	2,206.80	Gamma	(33)
Best supportive care	357.00	285.60	428.40	Gamma	(33)
Cost of drug administration per unit	17.00	13.60	20.40	Gamma	(33)
Proportion of receiving subsequent treatment					
Finotonlimab plus Bevacizumab	57.00%	45.60%	68.40%	Beta	(17)
Sorafenib group	68.10%	54.48%	81.72%	Beta	(17)
Cost of AEs, $					
Hypertension	35.46	28.37	42.55	Gamma	(31)
Proteinuria	105.57	84.46	126.68	Gamma	(31)
Decreased white blood cell count	454.26	363.41	545.11	Gamma	(34)
Platelet count decreased	1,505.92	1,204.74	1,807.10	Gamma	(34)
HFS	12.97	10.38	15.56	Gamma	(31)
Hypokalemia	3,000.00	2,400.00	3,600.00	Gamma	(34)
Utilities in finotonlimab plus bevacizumab group					
Utility of PFS	0.76	0.61	0.91	Beta	(35)
Utility of PD	0.68	0.54	0.82	Beta	(35)
Disutilities					
Hypertension	0.02	0.02	0.02	Beta	(36)
Proteinuria	0.20	0.16	0.24	Beta	(37)
Decreased white blood cell count	0.20	0.16	0.24	Beta	(34)
Platelet count decreased	0.20	0.16	0.24	Beta	(34)
HFS	0.15	0.12	0.18	Beta	(36)
Hypokalemia	0.04	0.03	0.05	Beta	(34)
Risk for main AEs in finotonlimab plus bevacizumab group					
Proteinuria	6.5%	5.20%	7.80%	Beta	(17)
Decreased platelet count	7.0%	5.60%	8.40%	Beta	(17)
Decreased white blood cell count	3.9%	3.12%	4.68%	Beta	(17)
Hypertension	8.7%	6.96%	10.44%	Beta	(17)
Hypokalemia	3.0%	2.40%	3.60%	Beta	(17)
Risk for main AEs in sorafenib group					
Proteinuria	0.9%	0.72%	1.08%	Beta	(17)
Decreased platelet count	2.6%	2.08%	3.12%	Beta	(17)
Decreased white blood cell count	4.3%	3.44%	5.16%	Beta	(17)
Hypertension	4.3%	3.44%	5.16%	Beta	(17)
Hypokalemia	2.6%	2.08%	3.12%	Beta	(17)
HFS	6.9%	5.52%	8.28%	Beta	(17)
Discount rate	5%	4.00%	6.00%	Beta	
Weight/kg	65				
$1 = ¥7.1217	40,354.27				

OS: overall survival, PFS: progression-free survival, PD: progression disease, AE: adverse event, HFS: palmar-plantar erythrodysaesthesia syndrome.

**Table 2:** Key clinical input data (US).

Parameters	Baseline value	Range		Distribution	Reference
		Minimum	Maximum		
Drug cost, $/per cycle					
Cost of *Finotonlimab*	8,640.00	6,912.00	10,368.00	Gamma	Estimated
Cost of *Bevacizumab*	8,270.47	6,616.38	9,924.56	Gamma	(38, 39)
Cost of Sorafenib	12,401.97	9,921.58	14,882.36	Gamma	(38, 39)
Cost of Lenvatinib	22,783.87	18,227.10	27,340.64	Gamma	(38, 39)
Cost of Atezolizumab	10,586.76	8,469.41	12,704.11	Gamma	(38, 39)
Cost of Regorafenib	23,298.13	18,638.50	27,957.76	Gamma	(38, 39)
Cost of Pembrolizumab	11,520.60	9,216.48	13,824.72	Gamma	(38, 39)
Cost of Tremelimumab	41,529.60	33,223.68	49,835.52	Gamma	(38, 39)
Cost of Durvalumab	12,582.45	10,065.96	15,098.94	Gamma	(38, 39)
Cost of Nivolumab	8,719.92	6,975.94	10,463.90	Gamma	(38, 39)
Cost of Ipilimumab	40,511.48	32,409.18	48,613.78	Gamma	(38, 39)
Cost of the laboratory test	111.65	89.32	133.98	Gamma	(40)
Enhanced CT	424.35	339.48	509.22	Gamma	(40)
Cost of end-of-life	21,603.00	17,282.40	25,923.60	Gamma	(24)
Best supportive care	965.19	772.15	1,158.23	Gamma	(41)
Cost of drug administration first hour	142.55	114.04	171.06	Gamma	(38, 39)
Administration intravenous, additional hour	30.68	24.54	36.82	Gamma	(38, 39)
Cost of AEs, $					
Decreased platelet count	22,698.00	18,158.40	27,237.60	Gamma	(42)
Decreased neutrophil count	17,181.00	13,744.80	20,617.20	Gamma	(42)
Hypertension	26,406.00	21,124.80	31,687.20	Gamma	(42)
Proteinuria	17,562.00	14,049.60	21,074.40	Gamma	(42)
HFS	28,405.00	22,724.00	34,086.00	Gamma	(42)
Hypokalemia	25,326.00	20,260.80	30,391.20	Gamma	(42)
Discount rate	3%	0.02	0.04	Beta	
Weight/kg	75				

AE: adverse event, BMI: body mass index, HFS: palmar-plantar erythrodysaesthesia syndrome.

### Model structure and analytical perspective

**Figure 1:**
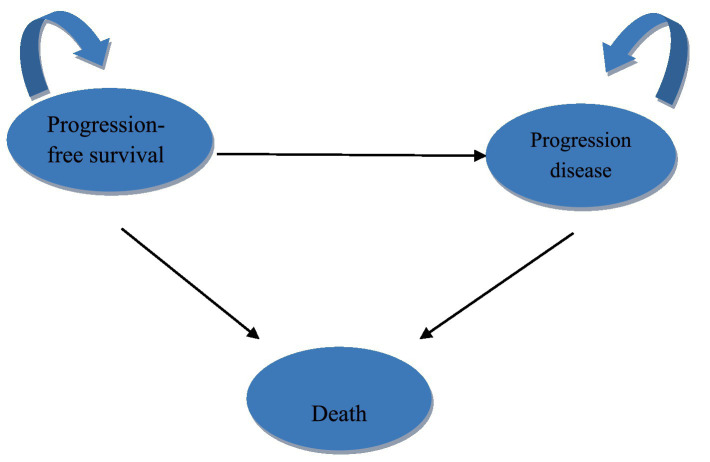
Markov model structure.

A state-transition Markov model with three mutually exclusive health states, PFS, progressive disease, and death, was developed using TreeAge Pro 2022 (Williamstown, MA, USA) and R software (version 4.2.4; Vienna, Austria). The model employed a 3-week cycle length and a 10-year time horizon, which were sufficient to capture more than 99% of lifetime mortality in the simulated cohort (Figure 1).

Analyses were performed from the healthcare payer’s perspective in both countries. The Chinese analysis adopted a national healthcare system perspective, whereas the U.S. analysis considered direct medical costs relevant to public and private payers (22).

### Model outcomes and economic parameters

Health outcomes were quantified in terms of quality-adjusted life years (QALYs). The economic results were summarized using incremental cost-effectiveness ratios (ICERs), incremental net health benefits (INHB), and incremental net monetary benefits (INMB). Consistent with country-specific pharmacoeconomic guidelines, both costs and health utilities are discounted annually at 5% in China and 3% in the U.S. (22, 23).

All cost inputs for China were converted to 2024 U.S. dollars using an exchange rate of $1 = ¥7.1217 and adjusted for inflation based on the national consumer price index. The WTP thresholds were defined as $40,354.27 per QALY in China (three times the national gross domestic product [GDP] per capita) and $150,000 per QALY in the U.S., reflecting commonly applied benchmarks from the World Health Organization and U.S. payer decision frameworks (24).

### Clinical effectiveness inputs

OS and PFS data were digitized from Kaplan–Meier curves reported in the phase 3 trial using the GetData Graph Digitizer software. Individual patient-level survival data were reconstructed using the algorithm proposed by Guyot and colleagues (25). Given the limited duration of trial follow-up, long-term survival was extrapolated over the full model horizon using parametric survival modeling (26).

The reconstructed time-to-event data were fitted to multiple candidate distributions, including exponential, Weibull, Gompertz, gamma, log-logistic, log-normal, and generalized gamma functions. The selection of the optimal survival model was guided by the Akaike information criterion, log-likelihood values, and visual comparison of fitted versus observed curves. The selected models were subsequently used to extrapolate the survival beyond the observed trial period (Supplementary Figures 1, 2).

Before applying Cox proportional hazards modeling, the proportional hazards assumption was evaluated through inspection of log–log survival plots and analysis of Schoenfeld residuals (27, 28). Although statistical testing suggested no significant violations (*p* > 0.05), visual assessment revealed potential departures from proportionality, including intersecting cumulative hazard curves and nonhorizontal residual trends. The predicted hazard variations across candidate distributions are illustrated in Supplementary Figures 3, 4, and the corresponding parameter estimates are summarized in Supplementary Table 1.

### Cost inputs

Direct medical costs incorporated into the model included drug acquisition, routine laboratory testing, contrast-enhanced computed tomography imaging, intravenous drug administration, subsequent systemic therapies, BSC, terminal care, and management of grade 3–4 adverse events. Drug prices in China were obtained from publicly available databases and institutional procurement schedules, while non-drug costs were sourced from previously published economic studies.

Because finotonlimab has not yet entered the U.S. market, its cost was approximated using the prices of comparable PD-1 inhibitors, specifically toripalimab and tislelizumab (29), as proxies. All cost inputs were standardized to 2025 U.S. dollars to facilitate cross-national comparisons. A summary of the clinical and economic parameters is provided in Tables 1, 2 (24, 30–42), respectively.

### Health utility inputs

Health state utility values ranged from zero (death) to one (perfect health). Utilities assigned to the progression-free and progressive disease states were 0.76 and 0.68 (35), respectively. Utility decrements associated with grade 3–4 adverse events were incorporated based on published estimates (Table 1) (34, 36, 37). For modeling simplicity, adverse events were assumed to occur during the first treatment cycle, with event probabilities derived from the trial incidence data.

### Price simulation analysis

Given the absence of an established U.S. price for finotonlimab, a price simulation analysis was conducted to identify the cost-effectiveness thresholds. The unit price of finotonlimab varied from $0 to $15,000 per dose, and ICERs were recalculated to determine the maximum price at which the regimen remained cost-effective under a WTP threshold of $150,000 per QALY.

### Scenario analysis

To assess the influence of post-progression treatment pathways on model outcomes, scenario analyses were performed by varying second-line therapy assumptions. Alternative treatment sequences were informed by the phase 3 trial protocol, national clinical guidelines (CSCO for China and NCCN for the United States), and expert consensus. In China, the evaluated strategies include PD-1 inhibitor monotherapy, targeted therapy, PD-1 inhibitor plus targeted therapy, PD-L1 inhibitor plus targeted therapy, and BSC. In the U.S., scenarios incorporate PD-1 inhibitors, PD-L1 inhibitors, targeted therapies, combinations of immunotherapy with targeted therapy or immunotherapy, and BSC.

A WTP threshold of $100,000 per QALY is also commonly used as a standard ICER cutoff in U.S. cost-effectiveness analysis (CEA) literature. Given that this cutoff would likely alter outcomes, base-case analysis, sensitivity analysis, and price simulation analysis were recalculated using this threshold.

### Sensitivity analysis

Uncertainty in model outcomes was examined using both one-way sensitivity analysis (OWSA) and probabilistic sensitivity analysis (PSA). In the OWSA, key parameters were varied individually by ±20% around their base-case values, and the resulting changes in ICERs were displayed using tornado diagrams to identify the parameters with the greatest influence.

For PSA, all model inputs were simultaneously sampled according to predefined probability distributions, with beta distributions applied to probabilities, utility values, and gamma distributions applied to cost parameters. A total of 10,000 Monte Carlo simulations were conducted to estimate the distribution of ICERs and the probability that finotonlimab plus bevacizumab would be considered cost-effective across a range of WTP thresholds.

## Results

### Base-case results

Across a 10-year analytic horizon, the base-case model estimated that treatment with finotonlimab plus bevacizumab generated 1.82 QALYs at a total cost of $36,669.98. In comparison, patients receiving sorafenib alone accrued 1.00 QALYs with an associated cost of $14,141.95. The combination regimen therefore produced an incremental benefit of 0.82 QALYs at an additional cost of $22,528.03, corresponding to an ICER of $27,505.33 per QALY (Table 3).

**Table 3:** The base case analysis.

Treatment	Cost	QALY	Incremental cost	Incremental QALY	INHB	INMB	ICER
Finotonlimab plus Bevacizumab (China)	36,669.98	1.82	22,528.03	0.82	0.26	10,523.82	27,505.33
Sorafenib (China)	14,141.95	1.00					
Finotonlimab plus Bevacizumab (US)	384,038.13	1.92	123,497.31	0.89	0.07	10,684.73	138,055.71
Sorafenib (US)	260,540.82	1.03					

QALY: quality-adjusted life year, ICER: incremental cost-effectiveness ratio, INMB: the incremental net monetary benefits, INHB: the incremental net health benefits.

From the Chinese healthcare system perspective, this ICER was substantially lower than the predefined WTP threshold of $40,354.27 per QALY. Under these conditions, the INHB was estimated at 0.26 QALYs, while the INMB reached $10,523.82, supporting the economic attractiveness of finotonlimab plus bevacizumab in China (Table 3).

When evaluated from the U. S. payer perspective, the ICER for finotonlimab plus bevacizumab was calculated to be $138,055.71 per QALY, remaining below the commonly applied U. S. WTP threshold of $150,000.00. The corresponding INHB and INMB were 0.07 QALYs and $10,684.73, respectively, indicating that the combination strategy is likely to be considered cost-effective under current U. S. pricing assumptions (Table 3).

### Price simulation analysis

**Figure 2:**
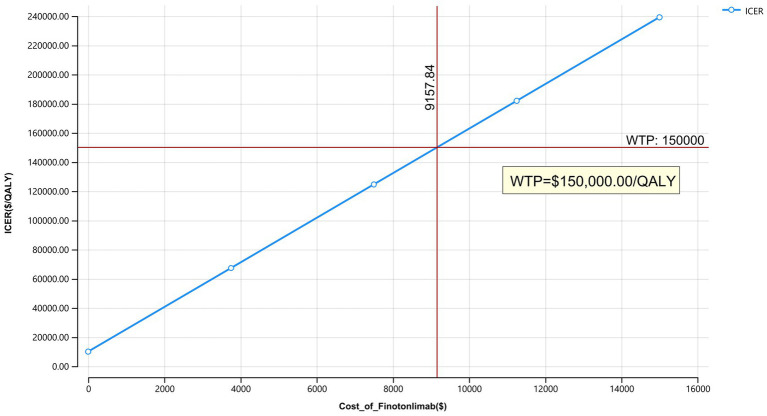
Price simulation of the U.S.

Figure 2 shows the results of the price simulation. As the assumed price of finotonlimab increased from $0 to $15,000 per 200 mg dose in the U.S. setting, the ICER values rose in a near-linear fashion. Based on the $150,000.00 per QALY WTP threshold, finotonlimab remained cost-effective when priced below $9,157.84 per 200 mg.

### Scenario analysis

Scenario analyses exploring alternative second-line treatment pathways are summarized in Supplementary Table 2. In China, none of the evaluated post-progression treatment scenarios altered the base-case conclusion, reflecting a wide margin between the estimated ICERs and the national WTP threshold. In the U.S. analysis, only the scenario assuming exclusive use of best supportive care following disease progression resulted in notable fluctuations; however, no alternative strategy fundamentally reversed the overall economic conclusion for the combination regimen.

When the U.S. WTP threshold was set to $100,000 per QALY, the ICER for finotonlimab plus bevacizumab remained $138,055.71 per QALY, which exceeded the threshold. Corresponding INHB and INMB were −0.34 QALYs and −$34,042.62, respectively, indicating that the combination strategy is unlikely to be considered cost-effective under this threshold. PSA results showed that at the $100,000 per QALY WTP threshold, finotonlimab plus bevacizumab was cost-effective in 26.69% of U. S. simulations. Based on the $100,000 per QALY WTP threshold, finotonlimab remained cost-effective when priced below $5,781.89 per 200 mg.

### Sensitivity analysis

**Figure 3:**
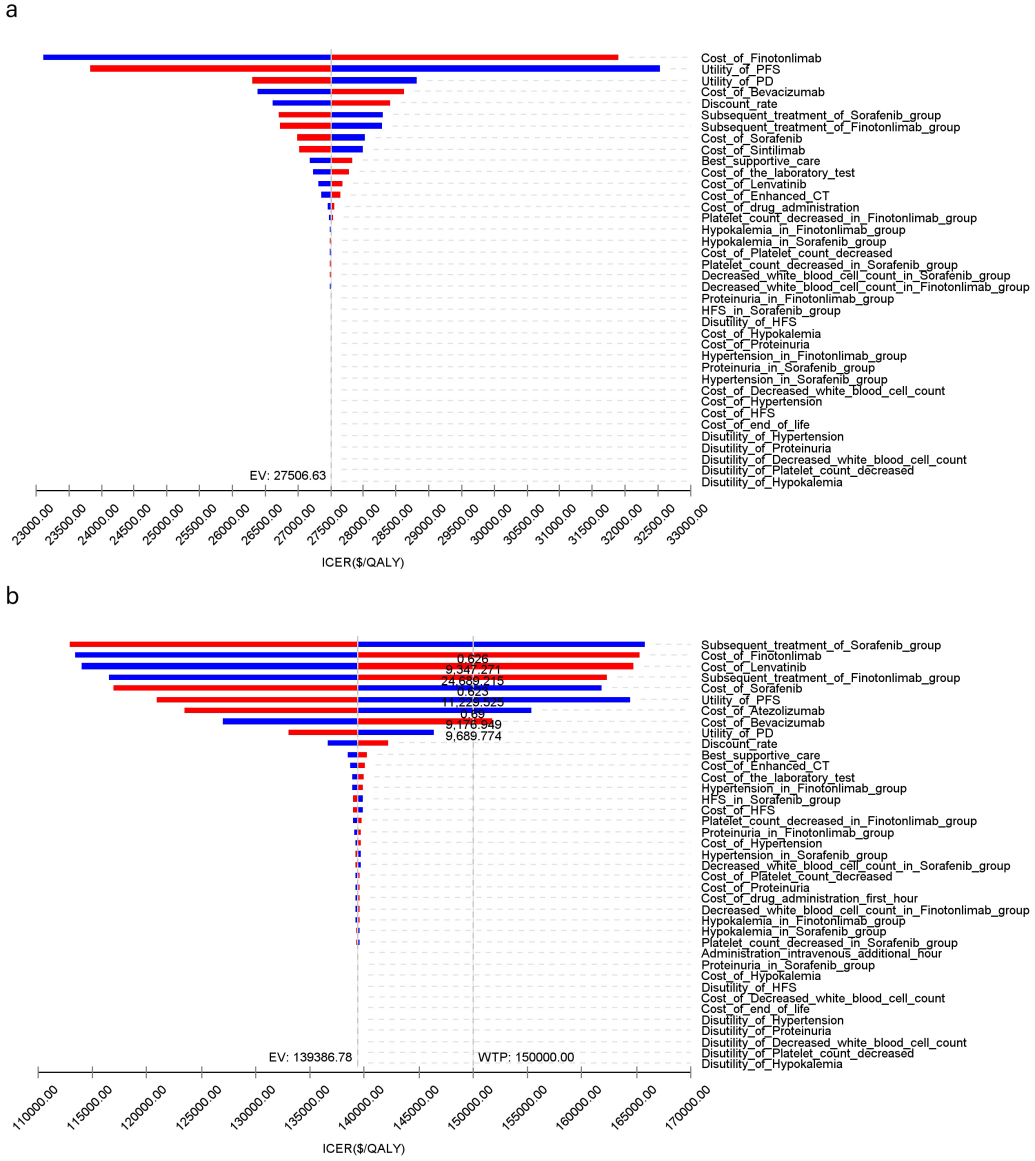
The tornado diagram of one-way sensitivity analysis: **(a)** China, **(b)** the U.S.

The OWSA results are presented as tornado diagrams in Figure 3. The red axes on the right half of the central axis indicate that higher values correspond to higher ICERs (lower cost-effectiveness), while blue axes indicate the opposite. Values on the horizontal axis represent ICERs exactly equal to WTP values. In the Chinese setting, the ICER was most sensitive to the acquisition cost of finotonlimab, followed by utility values assigned to progression-free and progressive disease states. In the U.S., the proportion of patients receiving subsequent therapy in the sorafenib arm, price of finotonlimab, and cost of lenvatinib exerted the greatest influence on ICER estimates (Figure 3). Owing to the relatively small difference in health outcomes between strategies in the U.S., variations in several high-impact parameters were sufficient to shift the cost-effectiveness conclusion. By contrast, no plausible parameter variation altered the conclusion in China.

**Figure 4:**
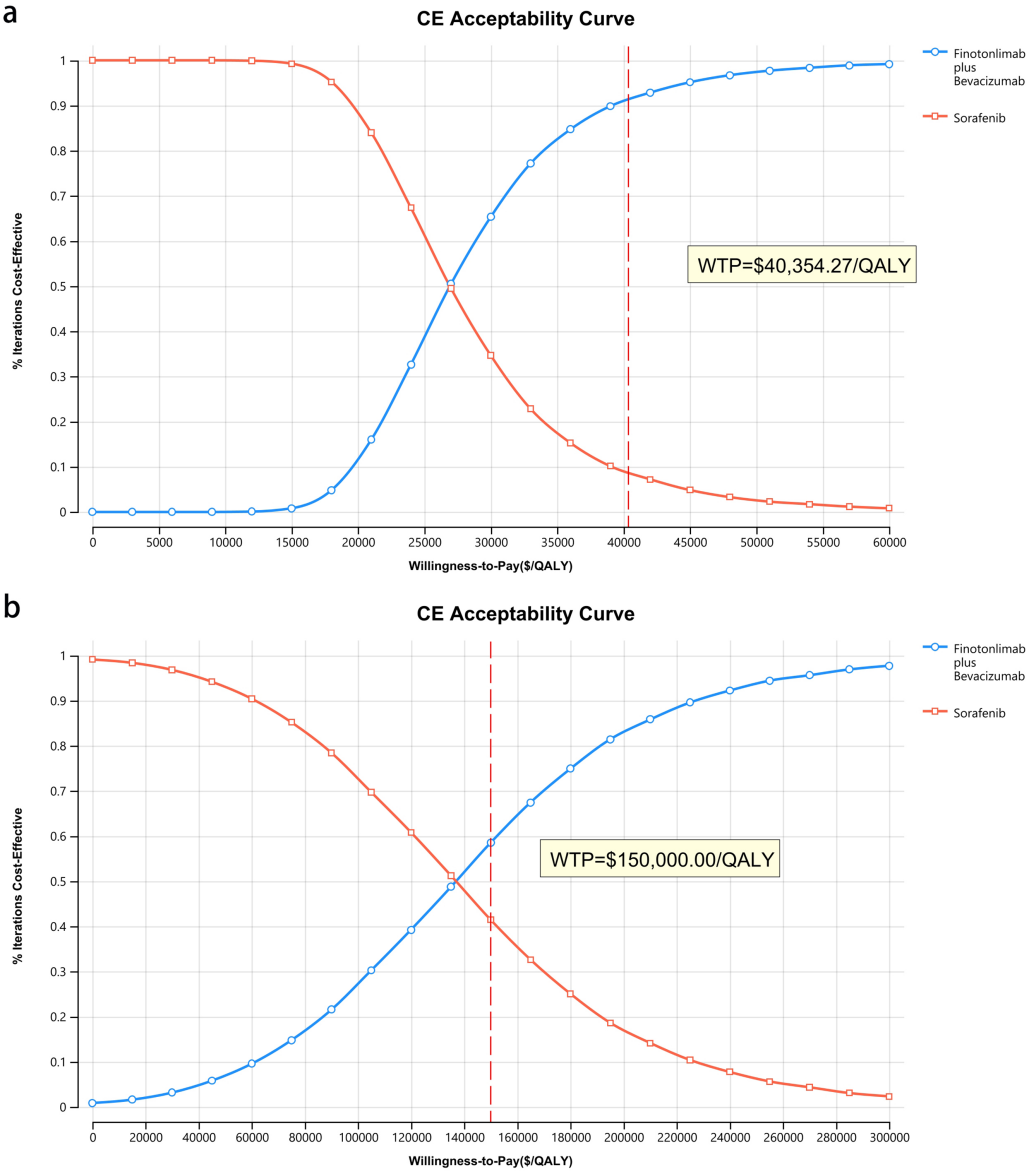
The cost-effectiveness acceptability curve: **(a)** China, **(b)** the U.S.

**Figure 5:**
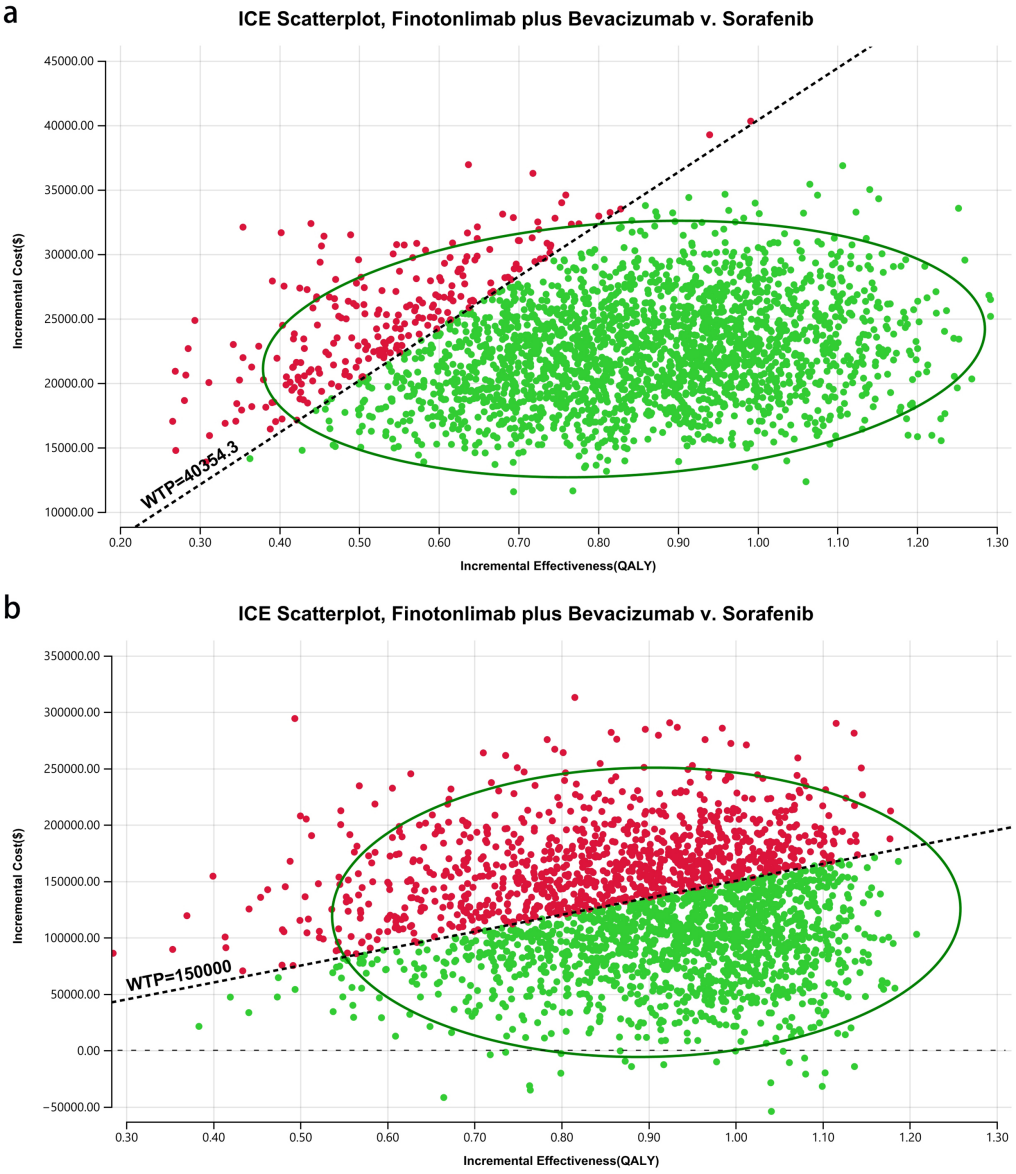
The cost-effectiveness probabilistic scatter plot: **(a)** China, **(b)** the U.S.

The PSA results are shown in Figures 4, 5, including the cost-effectiveness acceptability curve and the scatter plot. At the predefined WTP thresholds, finotonlimab plus bevacizumab was cost-effective in 91.43% of the simulations in China (three times GDP per capita; $40,354.27 per QALY) and in 58.33% of simulations in the U.S. ($150,000.00 per QALY).

## Discussion

The clinical efficacy and acceptable safety profile of finotonlimab in unresectable hepatocellular carcinoma provide a meaningful therapeutic advance for patients with limited treatment options (17). Building on this clinical foundation, the present study represents the first comprehensive economic evaluation comparing finotonlimab plus bevacizumab with sorafenib from both the Chinese healthcare system and U.S. payer perspectives. Under current pricing assumptions and commonly applied willingness-to-pay thresholds, the combination regimen was consistently associated with favorable cost-effectiveness outcomes in both settings. These findings address an important gap in evidence and help clarify the economic value of this novel treatment strategy. The high probabilities observed in the probabilistic sensitivity analyses further reinforce the stability of the base case results.

Previous cost-effectiveness studies examining first-line immunotherapy-based combinations for HCC have produced heterogeneous conclusions across regimens and health care systems. Several combinations, including anlotinib plus penpulimab (43, 44), sintilimab plus bevacizumab and camrelizumab plus rivoceranib (41, 45–47), have been reported to be cost-effective in China, whereas others, such as anlotinib plus penpulimab (48), atezolizumab plus bevacizumab (40, 49), and sintilimab plus bevacizumab have not (36). In the U.S., camrelizumab plus rivoceranib and durvalumab plus tremelimumab succeeded in meeting the cost-effectiveness thresholds (37, 41, 50, 51); however, atezolizumab plus bevacizumab was not considered cost-effective in multiple analyses (52). Against this backdrop of inconsistent findings, the present analysis yielded concordant conclusions in both China and the U.S. The incremental survival gains achieved with finotonlimab plus bevacizumab—0.82 QALYs in China and 0.89 QALYs in the United States—were sufficient to offset the relatively low treatment costs compared with other ICI-based regimens, resulting in favorable economic profiles across healthcare systems. From a patient-centered perspective, the availability of finotonlimab expands first-line treatment options and may alleviate therapeutic uncertainty for individuals with advanced HCC.

Sensitivity analyses provided additional insights into the drivers of economic value. In China, the substantial margin between the estimated ICER and national WTP threshold meant that plausible variations in key parameters did not alter the overall conclusion. Drug acquisition cost and health utility values for progression-free and progressive disease states emerged as the most influential inputs, highlighting the central role of pricing and quality-of-life outcomes in determining the value of innovative oncology therapies.

Although finotonlimab pricing has not yet been established in several international markets, the price simulation conducted in this study offers practical guidance for future reimbursement decisions. In the U.S., the analysis identified a pricing threshold below $9,157.84 per 200 mg as necessary for cost-effectiveness at a $150,000/QALY WTP benchmark. These findings may assist policymakers and payers in balancing affordability with access and highlight the importance of strategic pricing in maximizing the population-level benefits of ICIs in both China and the U.S.

The availability of multiple second-line treatment options for advanced HCC introduces additional complexity to economic modeling. In this study, post-progression treatment pathways were selected based on clinical guidelines, phase 3 trial protocol, and expert judgment to reflect clinically plausible practice patterns in each country. Although the model focused on second-line therapies and did not explicitly simulate later treatment lines, extensive scenario analyses were performed to assess the impact of alternative assumptions. Except for best supportive care–only scenarios in the U.S.–variations in subsequent treatment strategies did not materially change the study conclusions, indicating that the results are robust to reasonable differences in post-progression management.

This study has several limitations. First, while the underlying clinical trial was conducted across multiple centers in China (17), economic evaluations were extrapolated to both Chinese and U.S. healthcare systems, which may introduce uncertainty related to differences in patient characteristics, treatment response, and healthcare utilization. Second, the controlled setting and eligibility criteria of the randomized trials may limit the generalizability of the findings to routine clinical practice. Third, simplification of subsequent treatment sequences is necessary for model tractability, and real-world treatment pathways are often more complex. Despite these constraints, scenario and sensitivity analyses were performed to mitigate their potential impact. Overall, economic modeling based on robust trial data remains a valuable approach for informing policy and reimbursement decisions in oncology.

A additional limitation is that this study did not account for the management costs and disutility associated with grade 1–2 AEs. Given that the finotonlimab plus bevacizumab arm experienced a higher incidence of such AEs than the sorafenib arm, this oversight may underestimate the ICER. Additionally, the assumption that AEs only occur during the first cycle is unrealistic, as AEs can occur in any cycle and may persist for multiple cycles. This simplification likely underestimates costs and utility decrements, which could bias ICER estimates in either direction. The fact that the tornado diagram did not identify AE management as a notable factor on overall results may be attributed to the methodology used to determine AE management costs. Currently, apart from real-world research, there is no superior method to address this limitation, and this simplification is commonly adopted in pharmacoeconomic modeling. Future research may benefit from improved statistical methods, such as extending AE duration, refining cost estimates, or adopting alternative modeling approaches.

## Conclusion

Based on both base case and sensitivity analyses, finotonlimab in combination with bevacizumab demonstrates favorable cost-effectiveness compared with sorafenib as first-line therapy for unresectable hepatocellular carcinoma from the perspectives of the Chinese healthcare system and U.S. payers. Owing to differences in clinical data, costs, and modeling assumptions, the predictive power for the U.S. market is more limited. However, price simulation results suggest that a finotonlimab price below $9,157.84 per 200 mg in the U.S. is required to meet the conventional willingness-to-pay thresholds. These findings provide actionable evidence to support the pricing, reimbursement, and access decisions for this emerging immunotherapy-based combination.

## Data Availability

The original contributions presented in the study are included in the article/Supplementary material, further inquiries can be directed to the corresponding author.
