# Supplementary material for: Economic value of finotonlimab plus bevacizumab versus sorafenib for first-line treatment of unresectable hepatocellular carcinoma in China and the United States
Source: Front Public Health. 2026 Jul 6;14:1781790. doi: 10.3389/fpubh.2026.1781790 (PMC13381304; doi:10.3389/fpubh.2026.1781790)
Supplement: Supplementary file 1 [file Supplementary_file_1.DOCX]

**Supplementary Online Content**

**Economic Value of Finotonlimab Plus Bevacizumab Versus Sorafenib for First-Line Treatment of Unresectable Hepatocellular Carcinoma in China and the United States**

Wenwang Lang^1,4^, Qinling Jiang^2^, Xia Pan^2^, Lin Deng^2^, Xinrong Hu^2^, Zhijian Yang^3^, Yulong He^2^

1 Department of Pharmacy, Nanxishan Hospital of Guangxi Zhuang Autonomous Region, Guilin, China

2 Department of Oncology, Nanxishan Hospital of Guangxi Zhuang Autonomous Region, Guilin, China

3 Department of Hepatobiliary Surgery, Nanxishan Hospital of Guangxi Zhuang Autonomous Region, Guilin, China

4 Correspondence: Wenwang Lang, 290702062@qq.com 541002

**Supplemental Figure 1.** The Kaplan-Meier:(a) OS curves of Finotonlimab plus Bevacizumab group, (b) OS curves of Sorafenib group.

**Supplemental Figure 2.** The Kaplan-Meier:(a) PFS curves of Finotonlimab plus Bevacizumab group, (b) PFS curves of Sorafenib group.

**Supplemental Figure 3.** Comparison of Fitted Proportional Hazards Survival Models With Observed Kaplan–Meier Curves :(a) OS curves of Finotonlimab plus Bevacizumab group, (b) OS curves of Sorafenib group.

**Supplemental Figure 4.** Comparison of Fitted Proportional Hazards Survival Models With Observed Kaplan–Meier Curves :(a) PFS curves of Finotonlimab plus Bevacizumab group, (b) PFS curves of Sorafenib group.

**Supplemental Table 1**. The Akaike information criteria (AIC) and Bayesian information criteria (BIC)

**Supplemental Table 2.** The scenario analysis

This supplementary material has been provided by the authors to give readers additional information about their work.


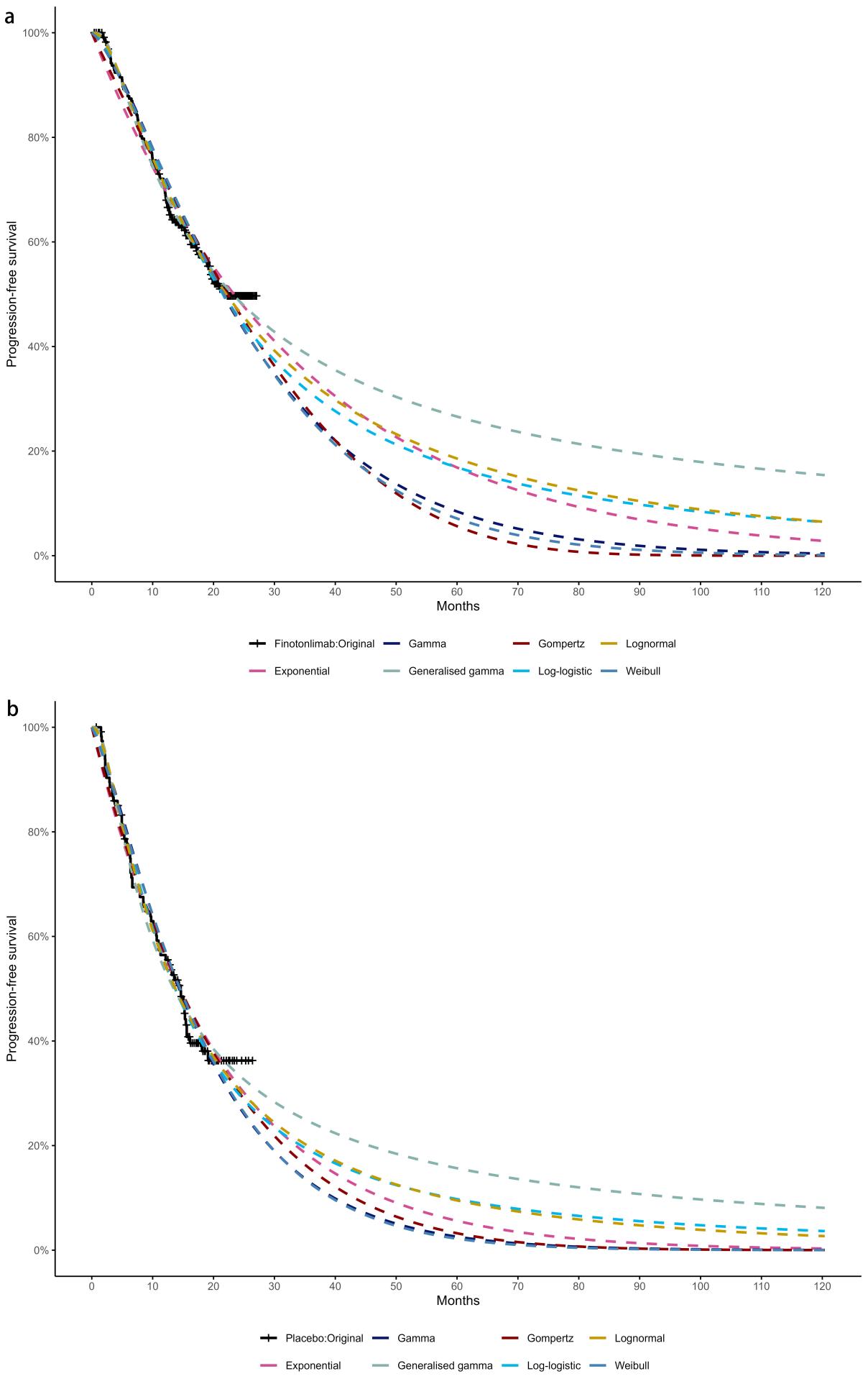


**Supplemental Figure 1.** The Kaplan-Meier:(a) OS curves of Finotonlimab plus Bevacizumab group, (b) OS curves of Sorafenib group.

**
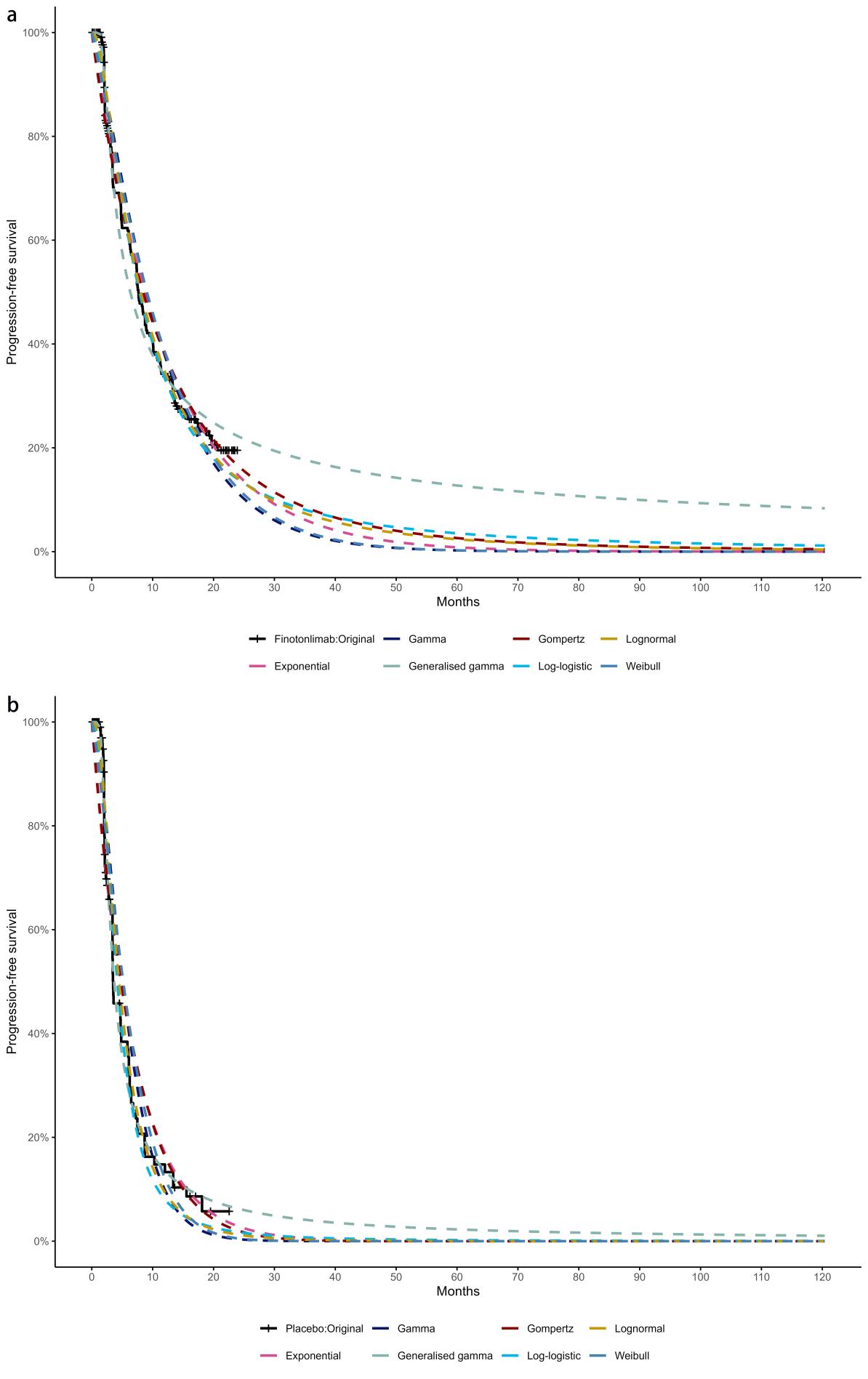
**

**Supplemental Figure 2.** The Kaplan-Meier:(a) PFS curves of Finotonlimab plus Bevacizumab group, (b) PFS curves of Sorafenib group.

**
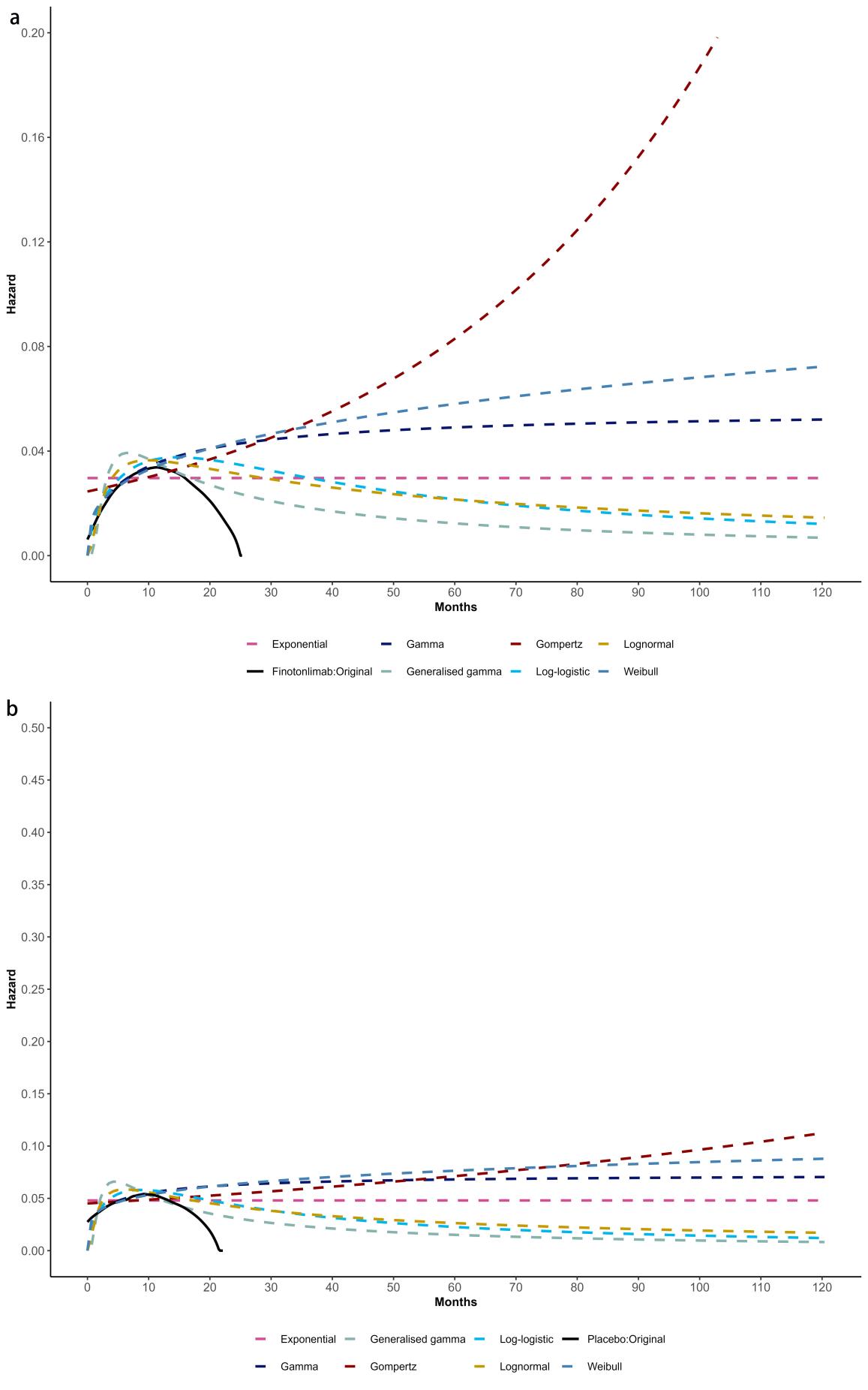
**

**Supplemental Figure 3.** Comparison of Fitted Proportional Hazards Survival Models With Observed Kaplan–Meier Curves :(a) OS curves of Finotonlimab plus Bevacizumab group, (b) OS curves of Sorafenib group.


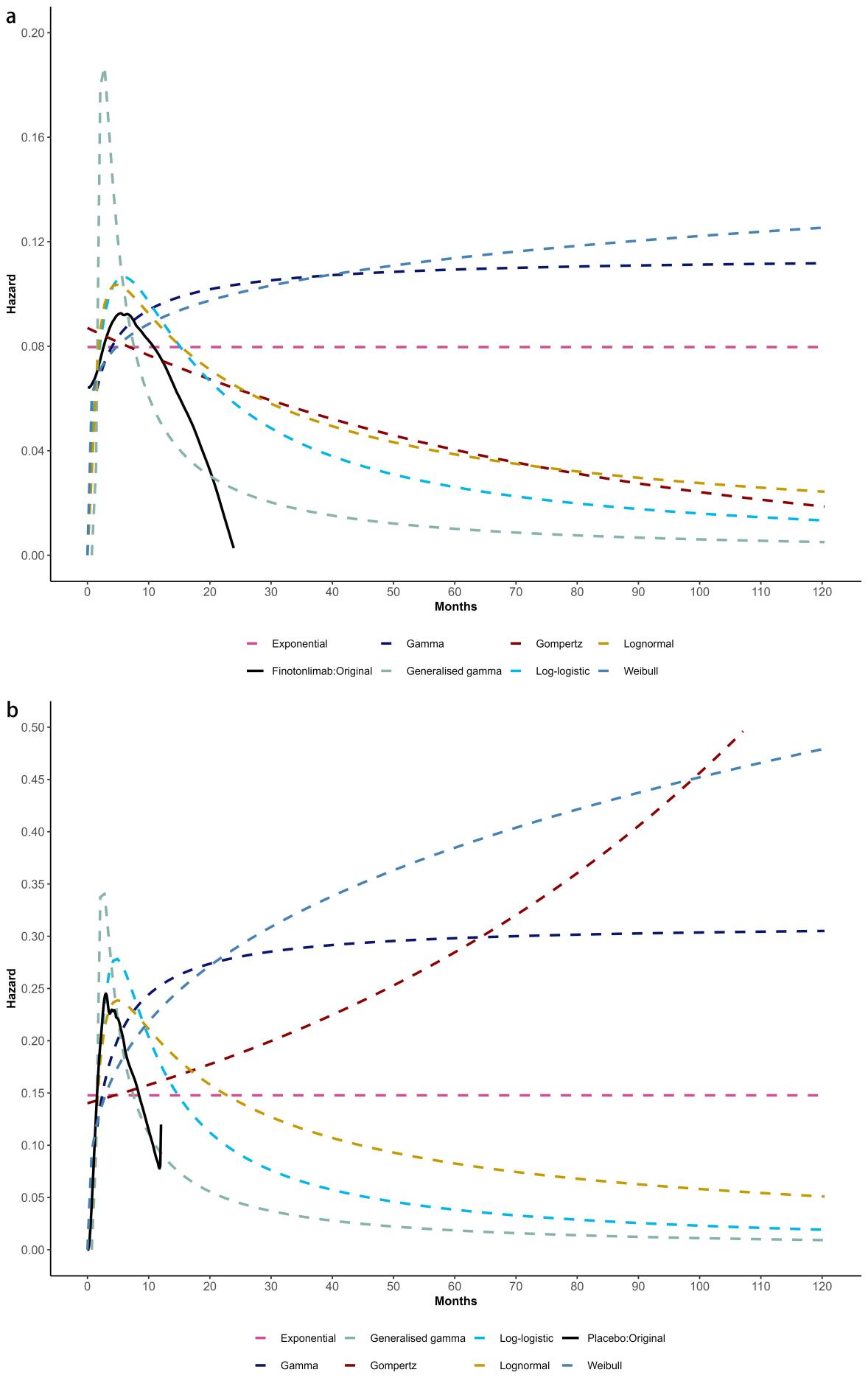


**Supplemental Figure 4.** Comparison of Fitted Proportional Hazards Survival Models With Observed Kaplan–Meier Curves :(a) PFS curves of Finotonlimab plus Bevacizumab group, (b) PFS curves of Sorafenib group.

**Supplemental Table 1**. The Akaike information criteria (AIC) and Bayesian information criteria (BIC)

| Type of distribution | Finotonlimab plus Bevacizumab (OS) | | | Sorafenib (OS) | | | Finotonlimab plus Bevacizumab (PFS) | | | Sorafenib (PFS) | | |
| --- | --- | --- | --- | --- | --- | --- | --- | --- | --- | --- | --- | --- |
|  | AIC | BIC | LogLik | AIC | BIC | LogLik | AIC | BIC | LogLik | AIC | BIC | LogLik |
| Exponential | 905.4830 | 908.9211 | -451.7415 | 534.7912 | 537.5447 | -266.3956 | 1067.9616 | 1071.3997 | -532.9808 | 409.7218 | 412.4754 | -203.8609 |
| Weibull | 899.0283 | 905.9045 | -447.5142 | 534.0574 | 539.5645 | -265.0287 | 1066.0795 | 1072.9557 | -531.0398 | 401.8603 | 407.3675 | -198.9301 |
| Gamma | 896.8484 | 903.7246 | -446.4242 | 532.8726 | 538.3798 | -264.4363 | 1061.5551 | 1068.4313 | -528.7776 | 394.0994 | 399.6066 | -195.0497 |
| Generalised gamma | 888.8824 | 899.1966 | -441.4412 | 527.6744 | 535.9352 | -260.8372 | 1010.8437 | 1021.1579 | -502.4218 | 355.6437 | 363.9045 | -174.8218 |
| Gompertz | 905.8829 | 912.7591 | -450.9415 | 536.6627 | 542.1699 | -266.3313 | 1069.1731 | 1076.0493 | -532.5866 | 411.5155 | 417.0227 | -203.7578 |
| Lognormal | 889.1214 | 895.9975 | -442.5607 | 527.0768 | 532.5840 | -261.5384 | 1033.3099 | 1040.1861 | -514.6550 | 374.2801 | 379.7873 | -185.1401 |
| Log-logistic | 893.8239 | 900.7000 | -444.9119 | 529.9545 | 535.4617 | -262.9773 | 1042.3751 | 1049.2512 | -519.1875 | 376.3416 | 381.8488 | -186.1708 |

OS：overall survival, PFS：progression-free survival, AIC：Akaike information criterion, BIC：Bayesian information criterion, LogLik：Log likelihood.

**Supplemental Table 2. The scenario analysis**

| **Treatment** | **INHB** | **INMB** | **ICER** | **PSA** |
| --- | --- | --- | --- | --- |
| Atezolizumab plus Bevacizumab(China) | 0.34 | 13645.84 | 23693.54 | 89.34% |
| Sintilimab plus Bevacizumab(China) | 0.16 | 6341.06 | 32612.22 | 80.77% |
| Tislelizumab(China) | 0.13 | 5231.77 | 33966.61 | 76.37% |
| Lenvatinib(China) | 0.12 | 4672.42 | 34649.53 | 74.65% |
| Regorafenib(China) | 0.11 | 4493.49 | 34868.00 | 73.73% |
| Best supportive care(China) | 0.10 | 4008.61 | 35460.01 | 71.31% |
| Atezolizumab plus Bevacizumab(US) | 0.20 | 29518.09 | 117002.18 | 72.53% |
| Pembrolizumab(US) | 0.11 | 16897.81 | 131110.21 | 64.57% |
| Durvalumab plus Tremelimumab(US) | 0.09 | 13313.372 | 135117.19 | 62.42% |
| Nivolumab plus Ipilimumab(US) | 0.13 | 19306.67 | 128417.37 | 66.65% |
| Regorafenib(US) | 0.25 | 36859.23 | 108795.64 | 73.70% |
| Lenvatinib(US) | 0.24 | 35974.62 | 109784.54 | 73.84% |
| Best supportive care(US) | -0.03 | -4616.95 | 155161.22 | 46.35% |

ICER: Incremental cost-effectiveness ratio, INMB：the incremental net monetary benefits, INHB: the incremental net health benefits, PSA: Probabilistic sensitivity analysis.
